# High-Speed Image Restoration Based on a Dynamic Vision Sensor

**DOI:** 10.3390/s26030781

**Published:** 2026-01-23

**Authors:** Paul K. J. Park, Junseok Kim, Juhyun Ko, Yeoungjin Chang

**Affiliations:** 1 Samsung Electronics, Hwaseong 18448, Gyeonggi-do, Republic of Korea; paulpark@gachon.ac.kr (P.K.J.P.); junseok7.kim@samsung.com (J.K.); juhyun03.ko@samsung.com (J.K.); 2 Department of Semiconductor Display, Gachon University, Seongnam 13120, Gyeonggi-do, Republic of Korea

**Keywords:** Dynamic Vision Sensor, CMOS image sensor, event-based vision, image restoration, motion blur

## Abstract

**Highlights:**

**What are the main findings?**

- We show that a Dynamic Vision Sensor (DVS), combined with a conventional image sensor and enhanced by event-driven techniques, can effectively suppress artifacts in motion blur compensation.
- We demonstrate that the proposed technique significantly improves the image quality of the blurred image.

**What are the implications of the main findings?**

- The event-based vision sensor can practically complement conventional CIS to achieve motion blur-resilient, high-speed imaging in smartphones without incurring prohibitive power or latency overhead.
- The demonstrated improvement under realistic low-illumination, fast-motion conditions suggests that future mobile camera designs can leverage DVS–CIS fusion as a viable system-level solution, rather than relying solely on heavier learning-based deblurring or more complex optics.

**Abstract:**

We report on the post-capture, on-demand deblurring technique based on a Dynamic Vision Sensor (DVS). Motion blur causes photographic defects inherently in most use cases of mobile cameras. To compensate for motion blur in mobile photography, we use a fast event-based vision sensor. However, in this paper, we found severe artifacts resulting in image quality degradation caused by color ghosts, event noises, and discrepancies between conventional image sensors and event-based sensors. To overcome these inevitable artifacts, we propose and demonstrate event-based compensation techniques such as cross-correlation optimization, contrast maximization, resolution mismatch compensation (event upsampling for alignment), and disparity matching. The results show that the deblur performance can be improved dramatically in terms of metrics such as the Peak Signal-to-Noise Ratio (PSNR), Structural Similarity Index Measure (SSIM), and Spatial Frequency Response (SFR). Thus, we expect that the proposed event-based image restoration technique can be widely deployed in mobile cameras.

## 1. Introduction

Recently, there has been a growing demand for high-speed and high-quality imaging in mobile devices, driven by the widespread use of smartphones as primary cameras in everyday life. Users expect to capture sharp images and videos of fast-moving scenes—such as children running, sports activities, or objects viewed from a moving vehicle—even under low-illumination conditions. However, motion blur caused by camera shaking and object motion remains a major obstacle to achieving such performance in compact handheld systems. In particular, when exposure time is increased to compensate for low light, motion blur becomes more severe, leading to a loss of fine texture, reduced contrast, and degradation of the overall image quality. Conventional approaches to mitigate motion blur in mobile cameras can be broadly classified into optical, algorithmic, and learning-based methods. Optical Image Stabilization (OIS) and advanced lens designs can reduce blur by mechanically compensating for hand tremors [1], but they have a limited effectiveness against complex object motion and are constrained by the size, cost, and power budgets of smartphone modules. Multi-frame fusion and deconvolution-based algorithms attempt to reconstruct a sharp latent image from a sequence of blurred frames [2], yet they often require accurate motion estimation and are sensitive to noise, rolling-shutter distortions, and saturation. More recently, deep neural networks have shown an impressive performance in image restoration [3], but their deployment on mobile platforms is challenging due to their high computational complexity, increased latency, and difficulty in guaranteeing robust behavior across diverse real-world conditions.

Event-based vision sensors, such as the Dynamic Vision Sensor (DVS), provide an attractive alternative to conventional frame-based imaging for addressing motion blur [4,5]. Instead of capturing full intensity frames at fixed intervals, a DVS outputs asynchronous events that represent temporal changes in logarithmic intensity at each pixel with a microsecond-level resolution. This sparse, high-temporal-resolution representation naturally encodes motion information and is inherently free from frame-integrated motion blur. As a result, the DVS has been actively explored for applications such as high-speed tracking, optical flow estimation, and event-based motion deblurring. When combined with a standard CMOS image sensor (CIS), the DVS can supply precise motion cues that guide the restoration of blurred intensity images. Nevertheless, integrating an event-based sensor with a conventional CIS in a mobile camera system is not straightforward. In practice, several non-idealities arise from differences in spatial resolution, dynamic range, temporal sampling, and optical alignment between the two sensors. These discrepancies can lead to characteristic artifacts in reconstructed images, including color ghosts around edges, residual blur in regions with low event activity, and noise amplification due to spurious events caused by sensor noise or background illumination changes. Furthermore, when the DVS and CIS observe the scene through different optical paths or with a baseline disparity, the naive fusion of their outputs can easily introduce geometric inconsistencies. To address these challenges, this work investigates a practical image restoration framework that combines a conventional CIS with a DVS for post-capture, on-demand motion deblur in mobile cameras. Building on a previous activity-driven and event-based restoration scheme [4], we systematically analyze the sources of artifacts that arise in a dual-sensor configuration and design event-driven compensation techniques tailored for realistic handheld scenarios. The proposed pipeline includes cross-correlation-based local threshold optimization to refine quantization error, contrast maximization for suppressing event noise while preserving informative structures, event-based resolution mismatch compensation (event upsampling for alignment) to partially compensate for resolution mismatch between CIS and DVS, and disparity- and timing-matching strategies to align the two sensor outputs in both space and time.

Recent studies on compensating CIS motion blur using an event camera (DVS) can be organized into two representative directions. The first direction focuses on maximizing restoration fidelity by adopting deep neural networks that fuse frame and event modalities [6,7,8,9,10,11,12,13]. While these learning-based methods achieve strong quantitative scores on standard benchmarks, they typically rely on relatively heavy feature extractors and GPU-oriented training/inference settings, which may impose non-trivial constraints for real-time deployment on mobile SoCs. The second direction originates from the physics/model-driven Event-based Double Integral (EDI) formulation, where events are used to relate a single motion-blurred frame to latent sharp images during the exposure interval [4]. Building on this idea, the multiple EDI further elaborates the event–frame imaging model and optimization procedure to improve robustness and reconstruction stability [14]. Subsequently, runtime-oriented variants have been proposed to push the EDI-style computation toward practical on-device use: Fast-EDI reports an implementation-level acceleration suitable for robotics/edge settings by optimizing the computational pipeline [15]. Furthermore, recent work introducing peripheral/trilinear EDI variants (e.g., P-TEDI) demonstrates that the core integral computation can be reorganized to significantly increase the event processing throughput and real-time factor [16]. Notably, model-based EDI-style reconstruction remains an attractive option for mobile deployment: while recent deep learning approaches often report Peak Signal-to-Noise Ratio (PSNR) values in the 35–36 dB range on the GoPro benchmark; EDI achieves around 30.29 dB under the same benchmark family, i.e., a smaller gap than might be expected given the large difference in computational requirements. Moreover, many learning-based pipelines implicitly assume GPU-class inference, whereas EDI-type formulations can be implemented efficiently on CPUs and have been further accelerated toward latency-tolerant processing. Motivated by this practicality, our work aims to make EDI-style CIS–DVS restoration robust on mobile devices by explicitly compensating the performance boundaries of the method under extreme deployment scenarios—cross-sensor mismatch (resolution/viewpoint/timing) and event noise-driven ghosting—which otherwise leads to a noticeable performance degradation in real CIS–DVS modules.

In addition to the algorithmic contributions, we place particular emphasis on quantitative evaluation under conditions that closely resemble practical smartphone usage. Using a Dead Leaves chart and Spatial Frequency Response (SFR) analysis, together with standard image quality metrics such as PSNR and the Structural Similarity Index (SSIM), we demonstrate that the proposed approach significantly improves the sharpness and fidelity of motion-blurred images captured under fast motion and low-illumination conditions. The overall restoration procedure is designed with mobile hardware constraints in mind, making it amenable to low-latency implementation on commercial smartphone platforms without incurring prohibitive computational overhead. The remainder of this paper is organized as follows. Section 2 explains the characteristics of the frame-based CIS and the event-based DVS. Section 3 describes the underlying principle of event-based image restoration and the issues encountered in mobile camera deployment, and Section 4 provides the evaluation methodology and metrics. The proposed compensation modules are then presented in Section 5, followed by conclusions in Section 6.

In this work, we focus on the practical integration of a mobile CIS–DVS dual-sensor system, where the main performance bottlenecks are often the sensor mismatch artifacts that appear in real devices. The key contributions are as follows:

- We clearly position the novelty as a mobile-ready integration and optimization of known event- and frame-based techniques, tailored to CIS–DVS co-sensing, with an explicit handling of real-device mismatch artifacts.
- We propose an edge cross-correlation-based local threshold optimization to suppress color ghost artifacts while preserving true motion edges.
- We introduce a lightweight contrast maximization (CM) method that reduces computation while maintaining robust event alignment under low illumination and sensor noise.
- We propose a lightweight alignment compensation for CIS–DVS discrepancies, including deterministic resolution mismatch compensation via bicubic resampling (upsampling) and disparity matching; this step is not computation-heavy and deep learning-based super resolution.
- We strengthen the evaluation by reporting complementary frequency-domain sharpness/blur metrics in addition to image quality metrics.

## 2. Frame and Event Based Sensors

This work is built on a practical hybrid sensing stack that combines a conventional frame-based CIS and an event-based DVS. The CIS provides dense intensity and color information at a fixed frame rate, while the DVS outputs asynchronous events only at pixels whose brightness changes exceed a threshold. Because motion blur mainly originates from temporal integration during CIS exposure and DVS events can preserve edge timing with a very low latency, their fusion is a natural fit for motion deblurring on mobile platforms.

### 2.1. Frame-Based CIS

In our prototype, the frame-based sensor is ISOCELL Bright GM1, a mobile CIS featuring a 12 Mp array (4000 × 3000) with 0.8 μm pixels in a 1/2″ optical format [17]. The sensor supports a 12 Mp output via a Tetrapixel (4-to-1) color filter arrangement and remosaic processing, enabling a higher sensitivity in low light and higher spatial detail in well-lit scenes. GM1 operates at 30 fps at full resolution and supports up to 120 fps at FHD and 240 fps at HD, which is useful for evaluating motion-dependent artifacts under realistic mobile capture modes. The shutter is an electronic rolling shutter with global reset; thus, temporal integration can still generate motion blur when the scene/camera motion is fast relative to the exposure time. From a system viewpoint, the CIS provides the absolute intensity and color (RGB) information required for photorealistic reconstruction and objective sharpness evaluation. However, its exposure-based sampling makes it vulnerable to blur and rolling-shutter distortions in dynamic scenes [18,19,20,21,22], which motivates the use of complementary event data for alignment and deblurring.

### 2.2. Event-Based DVS

The DVS used in this work has a 960 × 720 (≈0.7 Mp) resolution with a 4.95 μm pixel pitch. Unlike frame imagers, a DVS encodes changes in log-intensity as asynchronous ON/OFF events, which allows edge-like structures to be captured with a very high temporal resolution and without exposure integration [23,24,25,26,27,28]. In addition, the sensor integrates circuit- and readout-level techniques to reduce motion artifacts. A sequential column readout scheme is employed to avoid event-ordering artifacts, and a global event-holding function is introduced to suppress jello-like distortions in high-motion scenarios. Following the event-encoding architecture described in [29], we newly developed a dedicated DVS chip (RC1) tailored for mobile CIS–DVS integration. Specifically, while preserving the same fundamental pixel-level event generation principle, we reduced the sensor size and pixel resolution to satisfy mobile constraints for form factor, bandwidth, and power consumption. This mobile-oriented DVS design provides sufficiently dense edge/motion cues for fusion-based deblurring, while avoiding the unnecessary cost of high-resolution intensity imaging that is already provided by the CIS. At the device level, the DVS achieves a minimum speed of 2000 fps, highlighting its suitability for edge/temporal cue extraction on resource-constrained platforms when compared to continuously streaming full-frame intensity images.

As summarized in Table 1, the CIS and the DVS are complementary: the CIS carries dense intensity and color information but is susceptible to exposure-induced motion blur, whereas the DVS provides time-accurate edge cues without blur integration but lacks absolute intensity. In the remainder of this paper, we exploit this complementarity by aligning CIS edges and DVS events and by optimizing fusion parameters to maximize objective sharpness while suppressing event noise and mismatch artifacts.

## 3. Principles and Issues

### 3.1. Principle of Event-Based Image Restoration

Event cameras (i.e., DVS) measure brightness changes asynchronously at each pixel. Instead of integrating intensity over an exposure time, the sensor outputs ON/OFF events whenever the change in log-intensity exceeds a contrast threshold. Let *L*(*χ*,*t*) = log*I*(*χ*,*t*) denote the log-intensity at pixel *χ* = (*x*,*y*) and time t. An event *e_k_* = {*χ_k_*, *t_k_*, *p_k_*} is triggered when the temporal contrast satisfies the following:

Δ*L*(*χ_k_*, *t_k_*) = *L*(*χ_k_*, *t_k_*) − *L*(*χ_k_*, *t_k_^−^*) ≈ *p_k_C* (1)

where *p_k_* ∈ {+1, −1} indicates ON/OFF polarity and *C* > 0 is the event contrast threshold. As discussed in [4], this mechanism provides temporally precise measurements of intensity changes, which can be integrated to recover latent intensity evolution with minimal exposure blur. In contrast, a conventional CIS frame *B*(*χ*) is formed by exposure integration over time [*t*_0_,*t*_1_], which causes motion blur when the scene or camera moves:

(2) $$B\left(\chi \right)\approx \frac{1}{T}\int_{t_{0}}^{t_{1}}I\left(\chi ,t\right)dt,\;\;\;\;\;\;\;T=t_{1}-t_{0}$$

The key insight in [4] is that event streams can constrain the temporal evolution *I*(*χ*,*t*) during exposure, enabling deblurring by undoing the integration using event-driven intensity updates. For example, under the event generation model, the log-intensity can be expressed as an accumulation of events:

(3) $$L\left(\chi ,t\right)=L\left(\chi ,t_{0}\right)+C\sum_{t_{k}\in (t_{0},t]}p_{k}\delta (\chi -\chi_{k})$$

which implies

(4) $$I\left(\chi ,t\right)=I\left(\chi ,t_{0}\right)+exp\left(C\sum_{t_{k}\in (t_{0},t]}p_{k}\delta (\chi -\chi_{k})\right)$$

Substituting this equation into the exposure integration highlights how events provide a time-resolved modulation of intensity during the exposure. Following the principle in [4], one can construct a reconstruction that relates the blurred frame to the latent intensity at an exposure endpoint and the event accumulation between the endpoint and each time instant. This provides a model-driven (i.e., can be used for on-device mobile application) foundation for event-guided deblurring, where the recovered sharp image is consistent with both the CIS measurement (exposure integral) and the DVS measurement (asynchronous temporal contrast events).

### 3.2. Issues of Event-Based Image Restoration

**Figure 1:**
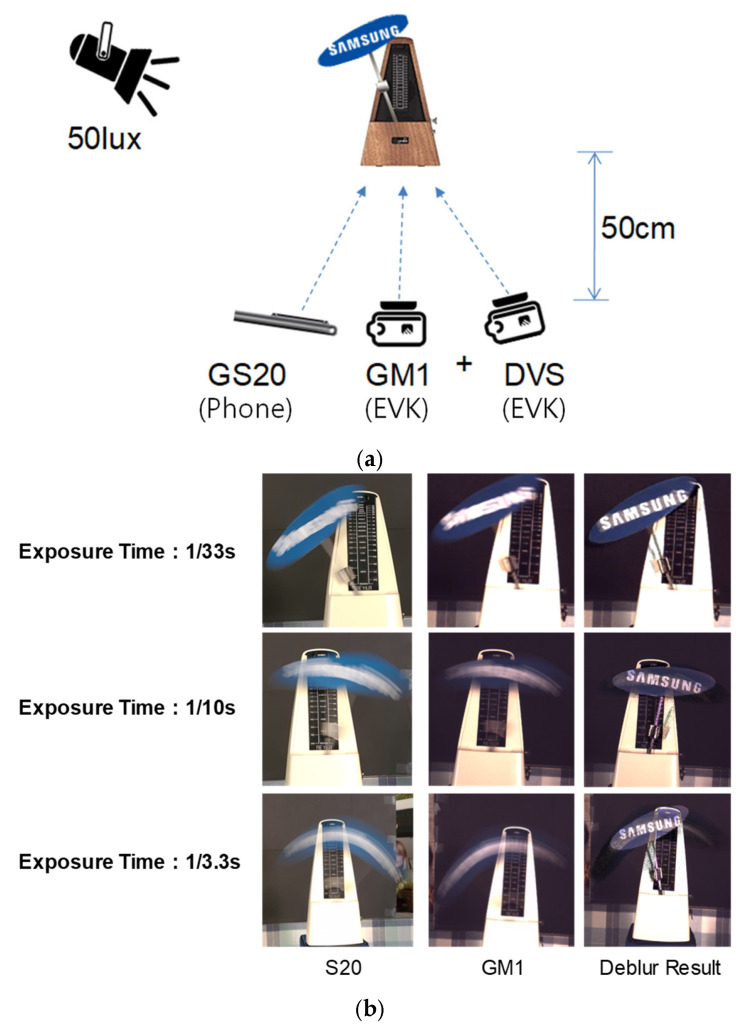
Motivation and baseline event-guided motion deblurring. (**a**) Experimental setup used to capture synchronized CIS frames and DVS events (50 lux illumination, 50 cm distance) using a commercial smartphone (Galaxy S20) and an external CIS (GM1) + DVS evaluation kit. (**b**) Motion blur examples at three exposure times (1/33 s, 1/10 s, and 1/3.3 s): conventional frame captures exhibit increasing blur with longer exposure, whereas the event-guided double-integral reconstruction [4] restores the metronome edges and the “SAMSUNG” logo at long exposure.

To motivate the use of events for motion deblurring in mobile camera imaging, we implement the baseline event-guided double-integral reconstruction proposed in [4]. Figure 1a shows the capture setup: a moving metronome with a “SAMSUNG” logo was recorded under approximately 50 lux illumination at a 50 cm distance. We captured conventional CIS images from a Galaxy S20 and, in parallel, a synchronized GM1 CIS stream, together with DVS events from evaluation kits. Figure 1b compares three exposure settings (1/33 s, 1/10 s, and 1/3.3 s). As exposure increases, frame-only CIS images exhibit progressively stronger motion blur, whereas the event-guided reconstruction restores sharper edges and improves the legibility of the logo even at long exposures.

This event-based approach is a suitable candidate for mobile camera applications because it can be implemented efficiently on mobile hardware (i.e., without heavy deep learning inference). However, we found that the reconstruction quality depends strongly on DVS characteristics (the event threshold and event noise) and on cross-sensor mismatch when combining CIS and DVS data (resolution, viewpoint/parallax, and timing). For example, the deblurring result in Figure 1b exhibits ghosting artifacts and distorted edge structures after blur compensation. These observations motivate the compensation modules introduced in Section 5: (i) cross-correlation-based local threshold optimization to stabilize the effective threshold selection, (ii) contrast maximization to suppress spurious events, and (iii) deterministic discrepancy compensation to align CIS–DVS representations on a common grid for robust fusion.

## 4. Evaluation Method

To evaluate the performance of motion blur compensation, PSNR and SSIM can be used. However, these metrics require a pixel-wise comparison between a sharp reference (ground truth) and the restored image. In this paper, we propose a simple evaluation metric based on a Dead Leaves chart and SFR. For example, the image blur can be described by using the transfer function *H*(*f*) in the frequency domain. Here, *f* denotes the spatial frequency (i.e., frequency in the image plane), not a temporal frequency. The spatial frequency is measured in cycles/pixel (or cycles/mm, depending on the calibration), and the spectral curves are obtained from the radially averaged spectrum.

(5) Y(f)=X(f)⋅H(f)

where *Y*(*f*) and *X*(*f*) are blurred and original images, respectively. *H*(*f*), the ratio of power spectral density (*ϕ*), can be defined as

(6) $$H\left(f\right)=\sqrt{\frac{{\left|\phi_{YY}\right|}^{2}}{{\left|\phi_{XX}\right|}^{2}}}$$

**Figure 2:**
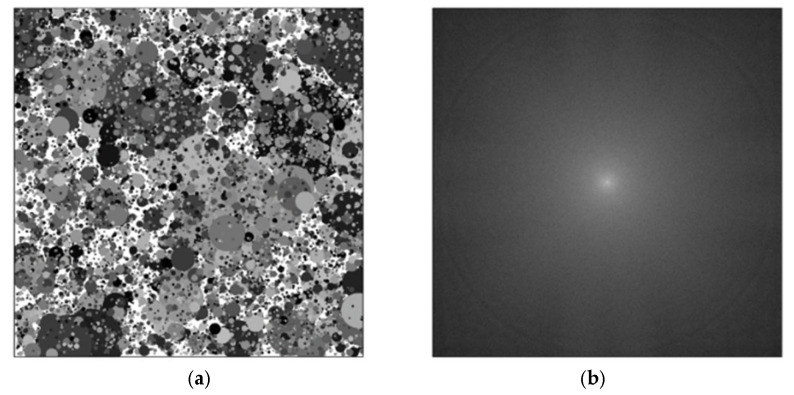
Frequency-domain evaluation based on Dead Leaves chart. (**a**) Dead Leaves chart. (**b**) Normalized radially averaged power spectrum (log scale) obtained from the 2-D FFT of (**a**) using spatial frequency coordinates (*f_x_*,*f_y_*) (cycles/pixel) and radial frequency *f* = √(*f_x_*^2^ + *f_y_*^2^).

For the frequency-domain analysis, we utilized a Dead Leaves chart as the evaluation chart. The Dead Leaves chart can be generated by allocating multiple circles whose radii and gray magnitudes are random values to random locations over the entire region, as shown in Figure 2a. Figure 2b is obtained from the 2-D Fourier transform of the Dead Leaves chart. Specifically, for an image *I*(*x*,*y*) of size *N_x_* × *N_y_*, we can compute the 2-D spectrum *F*(*u*,*v*) = *F*{*I*(*x*,*y*)} and the corresponding power spectrum *P*(*f_x_*,*f_y_*) = |*F(u*,*v*)|^2^. The spatial frequency coordinates are defined as *f_x_* = *u*/*N_x_* and *f_y_* = *v/N_y_* (cycles/pixel), which remain valid for rectangular images (*N_x_* ≠ *N_y_*). Because the Dead Leaves pattern is statistically isotropic, we can summarize the 2-D spectrum using a radially averaged profile *P*(*f*), where *f* = √(*f_x_*^2^ + *f_y_*^2^) denotes the radial spatial frequency. The power spectrum has no directionality due to the randomly allocated circles. In addition, it can be evenly distributed over the whole frequency domain due to various radii, as shown in Figure 2b. Thus, the Dead Leaves chart has been widely used for the evaluations of texture loss and sharpness because it is robust to scale change and invariant to rotation as well.

**Figure 3:**
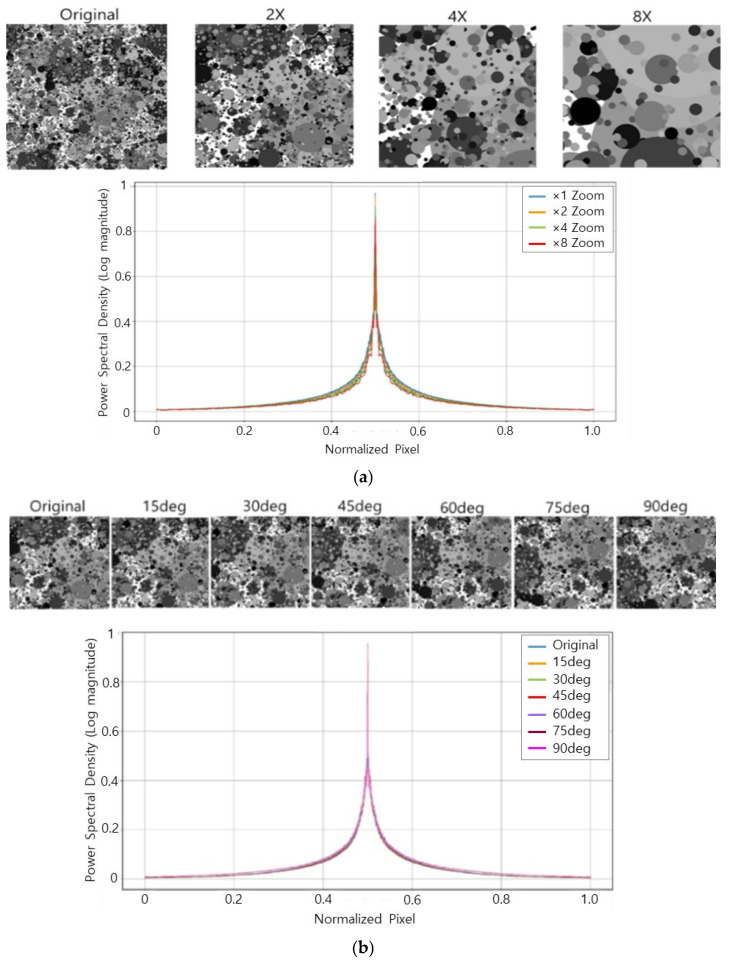
Frequency-domain robustness of the Dead Leaves chart used for SFR evaluation. (**a**) Scale invariance: normalized radially averaged log-magnitude spectra for different zoom factors (×2–×8) show negligible deviation. (**b**) Rotation invariance: spectra computed after rotating the chart (0–90°) overlap, confirming isotropy and direction-independent frequency content.

Figure 3 illustrates why the Dead Leaves chart is a reliable target for frequency-domain sharpness evaluation in our work. In Figure 3a (scale invariance), the same chart is rendered at different zoom factors (×2–×8) to mimic changes in the imaging distance. For each zoom factor, we compute the 2-D FFT of the chart, take the log-magnitude spectrum, and then obtain a 1-D power spectral density (PSD) profile through radial averaging (i.e., averaging spectral magnitude over all directions at the same spatial frequency). After normalizing each PSD by its low-frequency/DC energy, the spectra from different zoom levels almost perfectly overlap, indicating that the chart provides a multi-scale texture distribution that is robust to scale changes. Figure 3b (rotation invariance) repeats the same procedure after rotating the chart (0–90°). Because the chart is statistically isotropic, the 2-D spectra preserve circular symmetry and the radially averaged PSD curves again overlap. The small deviations that may appear near the Nyquist region are mainly due to discrete resampling during zoom/rotation, and they have a negligible impact on our subsequent MTF50-ratio measurement. These two invariance properties ensure that the SFR results reflect the effect of motion blur and restoration (rather than the chart’s orientation or scale), which is essential for fair comparisons across different motion directions and capture conditions.

**Figure 4:**
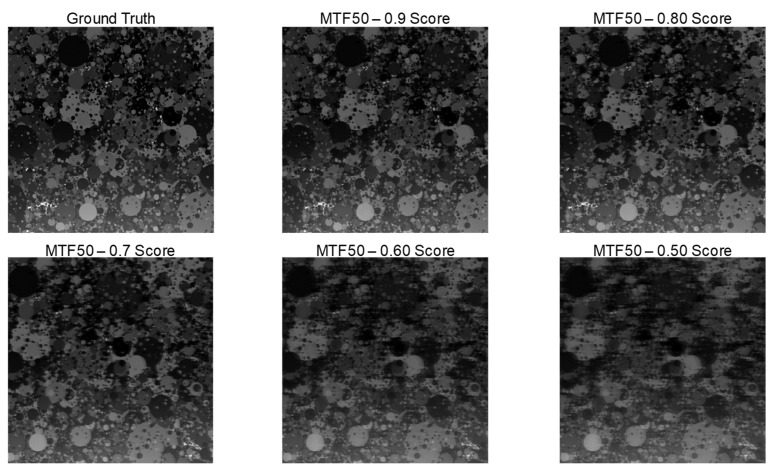
Relationship between motion blur severity and the proposed sharpness indicator (MTF50 ratio) measured from the Dead Leaves chart. To emulate different levels of motion blur, the chart was translated at multiple speeds while keeping the remaining capture conditions fixed. For each speed, the SFR was computed, and MTF50 was extracted as the spatial frequency at which the SFR drops to 50% of its low-frequency value. We report that the MTF50 ratio is measured from a sharp reference (ground truth) frame. As the chart speed increases, the effective motion becomes longer and the SFR is attenuated in mid-to-high frequencies, resulting in a decreased MTF50 ratio. Based on this empirical relationship, we adopt MTF50 ratio ≥ 0.7 as a practical criterion for “maintained sharpness” in the subsequent evaluations.

To quantify motion blur severity in a frequency-consistent manner, we use the MTF50 ratio derived from the SFR of the Dead Leaves chart. Specifically, MTF50 is defined as the spatial frequency at which the SFR falls to 50% of its low-frequency value, and it is widely interpreted as a compact indicator of effective resolvable detail [30,31,32]. In our evaluation, we normalize this quantity using a sharp reference frame (ground truth) and compute the ratio MTF50_blur_/MTF50_GT_. This normalization allows the metric to represent how much the mid-frequency contrast is preserved relative to an ideal/sharp baseline under the same optical and sampling conditions. For example, Figure 4 provides an empirical validation of this choice by sweeping the chart motion speed to generate progressively stronger motion blur. Figure 4 is based on simulation results in which the Dead Leaves chart is laterally translated at a constant velocity. Based on [33], the target was moved in the image plane (perpendicular to the optical axis) at speeds ranging from 0 to 1 m/s, with a camera-to-target distance of 1 m. As the motion speed increases, the exposure time integration corresponds to a longer effective motion, which acts as a stronger low-pass filter in the frequency domain. Consequently, the SFR drops earlier and the MTF50 value decreases, yielding a monotonic reduction in the MTF50 ratio. Importantly, this monotonic behavior makes the MTF50 ratio a practical and stable criterion for comparing blur levels and restoration gains. In our simulations, we observed that images with an MTF50 ratio below approximately 0.7 exhibit a visibly degraded texture/edge clarity, whereas ratios above this level generally maintain an acceptable sharpness. Therefore, we use an MTF50 ratio ≥ 0.7 as a conservative threshold for maintained sharpness and adopt the MTF50 ratio as the primary quantitative indicator in subsequent analyses.

In addition to MTF50, the integrated MTF area (MTF Integral) computed from the SFR curve can be utilized as an evaluation metric. Unlike MTF50, which captures a single point, the MTF area summarizes the overall preservation of contrast across spatial frequencies up to the Nyquist limit and can be computed directly from the existing SFR data. We can define the normalized MTF area as follows [30,31,32]:

(7) $$MTF\;area=\frac{1}{f_{N}}\int_{0}^{f_{N}}\phi \left(f\right)df$$

where *f_N_* is the Nyquist frequency. This integral metric is widely used in slanted-edge/SFR tasks. However, we observed that the MTF area may not align with a faithful restoration quality in the presence of the nonlinear edge enhancement introduced by motion deblurring. Specifically, deblurring may produce edge steepening and mild ringing/overshoot, which can inflate high-frequency MTF values beyond those of the ground truth (GT). As a result, the integrated MTF area can increase nonlinearly and even exceed the GT MTF area, which does not necessarily indicate an improved perceptual quality and may instead reflect over-sharpening artifacts. In contrast, the MTF50 ratio provides a more stable criterion for comparing how closely the restored image matches the GT under our objective of artifact suppression and faithful reconstruction. Therefore, we adopt the MTF50 ratio as the main quantitative metric.

As a complementary spatial domain metric that is directly sensitive to motion blur, we can compute the edge width from the Edge Spread Function (ESF), defined as the 10–90% rise distance (in pixels) of the normalized edge profile. The slanted-edge approach for estimating MTF from an edge profile was popularized for digital cameras and has been widely adopted and refined as part of International Organization for Standardization (ISO) 12233 [34,35]. The link between ESF broadening and MTF degradation is not merely empirical, but the MTF can be derived from an experimentally measured ESF by differentiating it into a Line Spread Function (LSF) and applying a Fourier transform [36]. In addition, fitting an analytical ESF model can improve the stability of the subsequent LSF/MTF estimation in the presence of sampling and noise [37]. In the mobile imaging context, it had been proposed that the ESF can be extracted on a per-frame basis to evaluate temporal MTF loss in smartphone slow-motion tasks, demonstrating a practical workflow for ESF-driven MTF evaluation [31]. For motion blur-specific evaluation, we can quantify high-speed motion blur using both Blurred Edge Width (BEW) and the MTF50 under ISO 12233-compliant measurements, and BEW increases while MTF50 decreases as motion blur becomes more severe [38]. Similarly, BEW can be analyzed as an objective blur metric for slow-motion/video-frame interpolation, with an emphasis on fast assessment without a dependence on GT data [39]. Moreover, it has been reported that a reduced MTF50 is accompanied by a broadened edge transition in practice [40]. Consistent with these studies, our motion blur simulation confirms that the ESF edge width decreases when the MTF50 increases, and we can use this metric as a supporting indicator of motion blur correction alongside the primary MTF50 results.

To strengthen real-world evaluation where a sharp reference is not available, the Variance of Laplacian (VoL) and Tenengrad gradient energy can be used as a blur measurement metric. Both operators have been extensively analyzed in the focus-measure literature and are known to increase as high-frequency content and edge contrast are restored [41,42]. This behavior is consistent with the optical interpretation of blur as a low-pass filtering effect. For example, the blur point-spread function attenuates high spatial frequencies in the MTF and therefore reduces spatial gradients and Laplacian responses. Accordingly, gradient/Laplacian-based blur scores tend to track MTF degradation under motion blur [38,43,44]. Specifically, VoL computes the variance of the discrete Laplacian response on luminance, while Tenengrad accumulates the squared Sobel gradient magnitude (i.e., higher values indicate less blur). The MTF50 value remains applicable regardless of ground truth availability—either against a sharp chart frame or against the input frame as a practical reference—and VoL/Tenengrad serve as additional reference-free indicators for in-the-wild demonstrations.

## 5. Results and Discussions

During the performance evaluation of event-based motion deblur, we found severe artifacts resulting in image quality degradation caused by color ghosts, event noises, and discrepancies between the CIS and DVS. To compensate for these artifacts, we propose and demonstrate event-based vision processing techniques based on cross-correlation optimization, contrast maximization, resolution mismatch compensation, and disparity matching.

### 5.1. Color Ghosts

**Figure 5:**
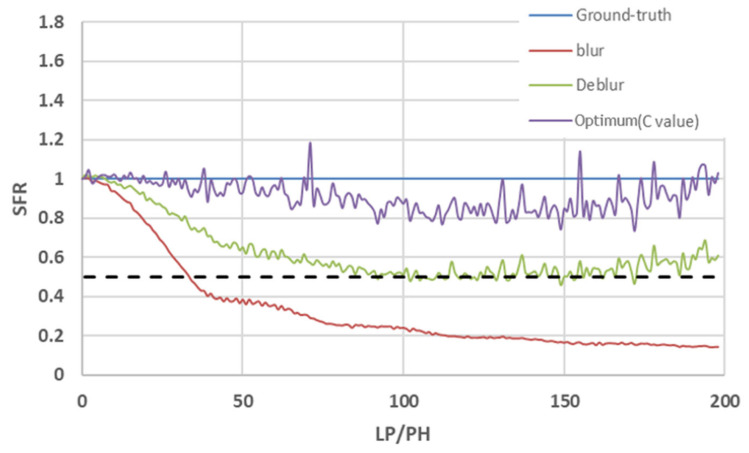
Frequency-domain validation of the cross-correlation optimization using the SFR framework. The plot reports the MTF50 ratio (normalized to the ground truth) over the LP/PH sweep for the ground truth sharp reference (blue), the motion-blurred input (red), the deblur output without the proposed local threshold optimization (green), and the output obtained using the c value selected by maximizing the (normalized) cross-correlation between the Sobel edge map of the deblurred frame and the event-derived edge map (purple). The horizontal dashed line denotes the 0.5 modulation level used to define MTF50. As LP/PH increases, the blurred input rapidly loses mid-frequency contrast and the MTF50 ratio drops, reflecting stronger motion blur. The baseline deblur partially restores MTF50, but its curve becomes unstable at a large LP/PH because motion deblurring can nonlinearly over-amplify high-frequency components (edge steepening and mild ringing), which does not correspond to a genuine increase in resolvable detail and can lead to an apparent high-frequency overshoot in frequency analysis. In contrast, the cross-correlation-optimized solution remains closest to the ground truth and achieves the highest MTF50 ratio throughout the sweep, experimentally confirming that maximizing edge cross-correlation is positively correlated with maximizing the MTF50 frequency. Importantly, this optimization acts as a lightweight control mechanism that suppresses high-frequency divergence while still improving mid-frequency contrast, thereby balancing sharpness enhancement and artifact suppression between CIS and DVS.

Each DVS pixel can generate an event when the log intensity reaches the threshold regardless of its scale (i.e., quantization error), which, in turn, causes a color ghost. We found that this ghost can also be generated due to the insufficient frame rate and response time of DVS. To mitigate the color ghosts, we utilize cross-correlation optimization based on a local thresholding method [4]. For example, blurred-pixel regions can be estimated accurately by integrating all event data weighted over exposure time. Thus, the edge region (*I_dvs_edge_*) can be defined as follows:

(8) $$I_{dvs\_edge}=\sum_{i=0}^{N}exp\left(-\frac{\left|{e(i)}_{ts}-f\right|}{T_{dvs\_fd}}\right)$$

where *N* is the number of events, *e*(*i*)*_ts_* is the event timestamp, and *T_dvs_fd_* is the average frame duration during the exposure time. Then, the optimized local threshold (*c*(*x*,*y*)) can be derived as follows:

(9) $$c\left(x,y\right)=\{\begin{array}{ll} argmax\left(I_{dvs\_edge}\left(x,y\right)\times I_{deblur\_edge\left(c\right)}\left(x,y\right)\right), & if\;I_{dvs\_edge}\left(x,y\right)\neq 0\\ argmax\left(-1\times I_{deblur\_edge\left(c\right)}\left(x,y\right)\right), & if\;I_{dvs\_edge}\left(x,y\right)=0 \end{array}$$

where *I_deblur_edge_*_(*c*)_ is the edge of the deblur image obtained after Sobel detection. For the restored CIS frame, *I_C_*, we can compute the Sobel gradients using the standard 3 × 3 kernels *K_x_* and *K_y_*. The horizontal/vertical gradients are *G_x_* = *I_C_* ∗ *K_x_* and *G_y_ = I_C_* ∗ *K_y_*, and the Sobel edge magnitude is defined as *E_Sobel_* = √(*G_x_*^2^ *+ G_y_*^2^). We then normalize *E_Sobel_* and use it as the frame-based edge representation in the cross-correlation term of (9), where it is compared with the event-derived edge map on the same spatial grid. This procedure follows the standard gradient-based edge extraction commonly used in the image processing literature [45]. Notably, since the objective operates on Sobel-derived edges, it is conceptually related to the Tenengrad gradient energy measure [41]. If the event-derived edge map acts as a mask, the correlation term can be viewed as an event-conditioned gradient energy accumulation, which we use as a lightweight cross-modal consistency cue for threshold optimization. Using this cross-correlation optimization, we can derive an appropriate threshold value and reduce color ghosts. Correlation-based objectives have been widely adopted for event-frame fusion and alignment. For example, the Event-based Double Integral (EDI) method integrates events to form an event-derived edge map and select the contrast threshold by maximizing the cross-correlation between Sobel edge maps of the reconstructed latent image and the event edge map [4]. In stereo hybrid event–frame sensing, an edge-based disparity estimator was proposed to compute cross-correlation between edges extracted from the event stream and edges detected in the frame data within a coarse-to-fine framework [46]. More generally, it has been reported that (normalized) cross-correlation can be directly optimized in a least-squares form for image alignment, motivating correlation-driven optimization under contrast/illumination changes [47]. Figure 5 shows the estimated SFR when Line Pairs/Picture Height (LP/PH) is increased. The results show that the proposed cross-correlation optimization technique can obtain clear edges (less ghosts) by optimizing the local threshold. Figure 5 empirically validates this correlation-based optimization. The local threshold selected by maximizing the edge cross-correlation consistently yields the highest MTF50 ratio across the LP/PH sweep, indicating that the correlation objective is strongly aligned with maximizing the MTF50 value (as described in Section 4). We also observed that aggressive motion deblurring alone can nonlinearly amplify unnecessary high-frequency components (edge steepening/ringing), which may cause frequency-domain measures to behave unstably. Thus, we conclude that the cross-correlation selection suppresses such divergence while preserving the mid-frequency contrast gain.

### 5.2. Event Noise

The DVS output also includes a significant number of spurious events that do not correspond to meaningful scene motion. These event noises originate from several sources, such as sensor dark current, junction leakage, background illumination flicker, and random fluctuations around the contrast threshold. In addition, a slight mechanical vibration of the setup and electronic readout noise can trigger events even in visually static regions. When these noisy events are integrated to reconstruct an intensity image, they behave similarly to high-frequency speckle or texture-like artifacts and directly degrade the performance of motion deblur. In particular, we observed that noise events tend to accumulate in low-texture areas and around weak edges, where the true number of motion-induced events is relatively small. As a result, the deblurred image exhibits residual graininess and irregular edge profiles, which in turn limits the achievable improvement in PSNR and SSIM. To reduce these event noises, we applied the contrast maximization (CM) method, which can filter out inconsistent events by maximizing image contrast after warping spatiotemporal events [48]. In the CM framework, the raw event stream is first collected over a short time window that covers the exposure interval of the CIS frame. Then, a simple motion model is assumed for each local region (for example, linear translation along the dominant motion direction), and the events are warped along candidate motion trajectories onto a 2-D accumulation plane. If the assumed motion is correct and the events are mostly signal-dominant, the warped accumulation forms a sharp, high-contrast edge pattern. In contrast, noise events that are not coherent with the local motion hypothesis are dispersed over the plane, leading to a smoother and lower-contrast image. By searching for the motion parameters that maximize the contrast of the accumulated events, we can implicitly suppress spatially and temporally inconsistent noise events.

**Figure 6:**
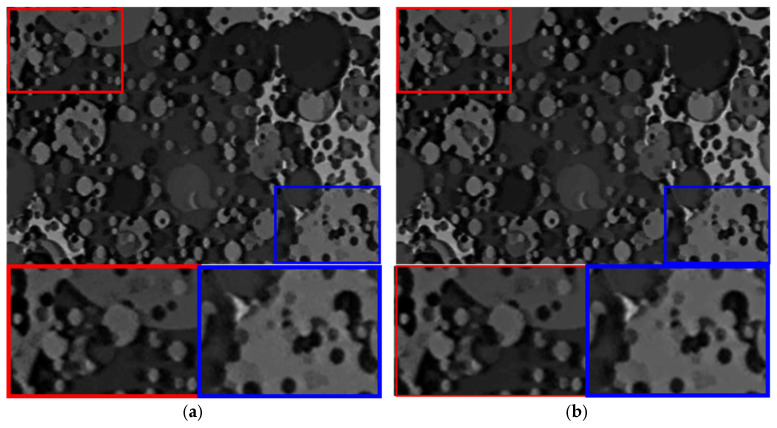
Effect of CM-based event noise filtering on event-based motion deblurring. (**a**) Deblurred result reconstructed using the raw (unfiltered) event stream, where spurious events tend to accumulate during double integration and appear as fine-grain speckle/texture-like artifacts, potentially perturbing weak edges and low-texture regions. Representative regions of interest (ROIs) are indicated by colored boxes, and the corresponding magnified crops are shown for closer inspection. (**b**) Deblurred result after applying the CM, where events inconsistent with the dominant local motion hypothesis are suppressed by selecting the motion candidate that maximizes a variance-based contrast measure. The filtered reconstruction becomes visually smoother in homogeneous areas while preserving prominent edge sharpness, yielding slight but consistent improvements in PSNR and SSIM (reported under each panel). This filtering step also helps stabilize subsequent stages by preventing noise accumulation in later super-resolution processing. To further visualize the speckle-like nature of event noise and the effect of the proposed filtering, pseudo-color ROI views are provided in the Supplementary Materials (Figure S1).

In our implementation, we adopted a lightweight CM formulation tailored for low-latency operation on a mobile platform. The event stream was divided into small tiles, and a limited set of motion candidates was evaluated per tile to bound the computational cost. For each candidate, events were warped and accumulated into an intermediate image, and a simple contrast metric—based on the variance of intensity values within the tile—was computed. Only the events associated with the best-contrast candidate were retained for subsequent double integration, while the others were discarded as noise. This local CM-based pruning significantly reduces the density of noisy events without requiring explicit per-event classification or heavy learning-based models. Figure 6 compares the motion deblur performance before and after applying CM-based event noise filtering. As shown in Figure 6a, the deblurred images without noise filtering contain noticeable fine-grain artifacts and slightly distorted edges, especially in regions with low texture. After CM is applied, Figure 6b demonstrates that the reconstructed images become visually smoother in homogeneous areas while preserving the sharpness of prominent edges. Quantitatively, the SSIM and PSNR are improved slightly but consistently across the tested scenes. Although the numerical gain appears modest, this step is crucial for stabilizing the overall restoration pipeline because it prevents noise accumulation in the subsequent cross-correlation optimization and super-resolution stages. Therefore, CM-based event noise filtering plays an important supporting role in achieving robust and perceptually pleasing motion deblur performance in the proposed technique.

### 5.3. Discrepancies

**Figure 7:**
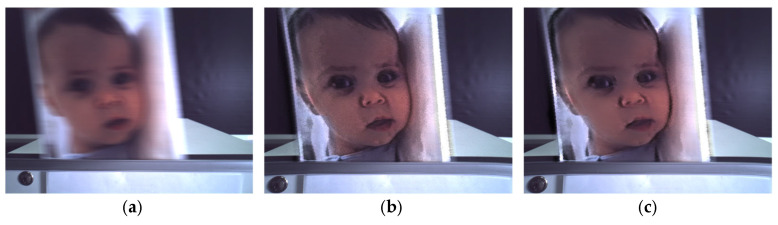
Effect of CIS-DVS discrepancy compensation on a real mobile dual-sensor module. (**a**) Motion-blurred CIS input. (**b**) Event-based deblur result (double-integral reconstruction) without discrepancy compensation, showing residual edge doubling and jitter caused by (i) spatial resolution mismatch between CIS frames and the DVS event grid, (ii) temporal mismatch due to CIS exposure/rolling-shutter readout versus asynchronous event timestamps, and (iii) residual geometric misalignment (parallax). (**c**) Final restoration after applying the proposed lightweight compensation modules (bicubic upsampling of the event representation to the CIS grid, row-wise timing correction for rolling-shutter exposure, and disparity/edge correlation alignment), resulting in improved edge coherence and reduced residual artifacts.

The deblur performance can also be degraded by discrepancies between the CIS and DVS that exist in real mobile devices. In our CIS–DVS module, we observed three dominant mismatch factors: (i) a spatial resolution mismatch between CIS frames and the DVS event grid, (ii) a temporal mismatch caused by CIS exposure/rolling shutter and the asynchronous event timestamps, and (iii) a residual geometric misalignment (parallax) due to the physical baseline and optics. If untreated, these mismatches lead to edge doubling, local blur, and jitter in the restored image, as shown in Figure 7b.

First, to compensate for the spatial mismatch, it is necessary to increase the DVS resolution. Basically, the proposed super-resolution refers to deterministic resampling that maps the event representation onto the CIS sampling grid for alignment; it is not a deep learning-based super-resolution that hallucinates new textures. We upsampled the event edge/event-frame representation to the CIS resolution using bicubic interpolation, which is computationally inexpensive and sufficiently accurate because the events are used as motion/alignment cues while the final texture is provided by the CIS image. Bicubic interpolation is implemented as separable cubic convolution in the horizontal and vertical directions, following the standard formulation in [33,49]. In this work, we tested several interpolation techniques including linear, bilinear, bicubic, and Gaussian. As a result, the bicubic method showed the best performance. This was because the super-resolution could be achieved by spreading the temporal events over 3-D space, and the bicubic method produced events with less blurring and fewer artifacts during upsampling, which in turn retained complex edges and structural details, leading to a better visual quality and higher objective metrics (PSNR and SSIM). Recent event-guided super-resolution methods demonstrate an impressive reconstruction quality by using deep networks and learned fusion/alignment modules [50,51,52,53,54]. However, these approaches typically require non-trivial compute/memory training data matched to the specific CIS–DVS hardware and can suffer from domain shift and hallucinated textures under real mobile noise and High-Dynamic-Range (HDR) conditions. Our goal is robust, on-device processing with a minimal latency and predictable behavior. Therefore, we intentionally adopted a lightweight, model-free alignment compensation (bicubic resampling + disparity matching) that can be implemented efficiently on a smartphone-class ISP/SoC. To account for the small viewpoint difference between CIS and DVS, we calibrated a global (or piecewise) displacement using edge–space correlation and compensated it before fusion. This improves local consistency around high-contrast boundaries and reduces residual ghosting, especially in scenes with depth variation.

In the experiment, the resolution and shutter control of our GM1 were 12 Mp and rolling-based, respectively. On the other hand, our DVS had a 0.7 Mp resolution and global shutter operation, as described in Table 1. In this case, the subject image was moved from left to right to cause the motion blur in front of the CIS-DVS module. The shutter mismatch could also be compensated by adjusting the pixel readout time line by line. Consequently, Figure 7c shows reduced edge doubling and an improved stability compared with the uncompensated result in Figure 7b.

**Figure 8:**
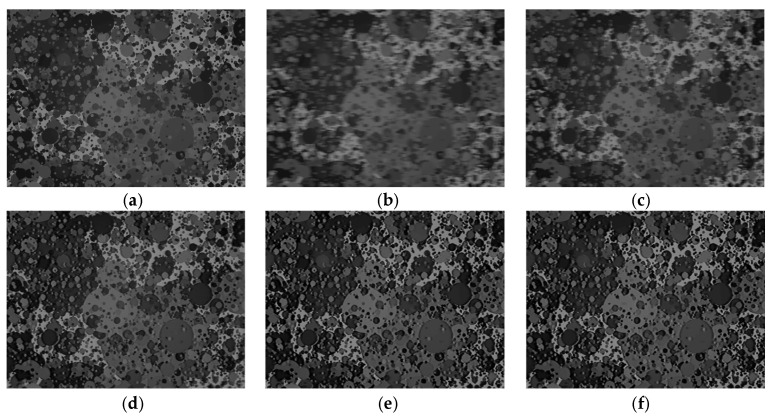
Step-by-step ablation results of the proposed techniques for the Dead Leaves chart. (**a**) Ground truth reference. (**b**) Motion-blurred CIS input. (**c**) Baseline event-guided motion deblurring using the double-integral reconstruction. (**d**) Deblurring with the proposed cross-correlation optimization. (**e**) Result after resolution-mismatch compensation. (**f**) Final restoration result after the noise filtering. Overall, the sequential activation of the proposed modules increases texture fidelity and edge coherence while reducing artifact/noise, which is reflected by improved PSNR/SSIM with respect to the ground truth reference.

To validate the proposed cross-correlation optimization, contrast maximization, resolution mismatch compensation, and disparity matching techniques quantitatively, we measured the performances using the moving Dead Leaves chart. Figure 8 presents the restoration results obtained from a simulation. The simulation procedure is identical to that used for Figure 4 (based on [33]). Specifically, we generate motion blur by translating the target image with a constant velocity in the image plane (i.e., perpendicular to the optical axis), while keeping the camera-to-target distance fixed at 1 m. In this experiment, the translation speed is set to 1 m/s to represent a severe fast-motion condition. In this case, the DVS frame rate was set to 2000 fps. Figure 8 and Table 2 show the results obtained by incrementally applying the proposed techniques. Note that the PSNR, SSIM, and MTF50 ratio of the blurred input image (Dead Leaves chart) were 18.52 dB, 0.683, and 0.39, respectively, and these metrics improved to 38.72 dB, 0.911, and 0.99 in the final output.

We confirmed that the proposed method can be executed on-device in a post-capture motion blur improvement. The current prototype implementation runs on the CPU and requires approximately 8 s per frame. We clarify that this latency is not intended to meet live video-rate processing (e.g., 30 fps), but rather targets a common mobile user scenario: after capture, a user reviews the saved photo and triggers an on-demand deblurring function only when noticeable motion blur is present; under this interactive workflow, we think a single-digit-second latency can be acceptable. Due to OS-level scheduling and the camera/event driver pipeline, it is difficult to obtain cycle-accurate module-wise timing; nevertheless, coarse profiling consistently indicates that the cross-correlation-based local threshold optimization is the dominant computational component, while the remaining steps (noise reduction, event resampling for alignment, and fusion) contribute comparatively less. Because the cross-correlation optimization is highly parallelizable, we expect that exploiting mobile accelerators (GPU/NPU/ISP) can substantially reduce the processing time in future deployments. Thus, these results support the practical applicability of the proposed lightweight design for smartphone deployment.

## 6. Conclusions

In this work, we demonstrated that combining a conventional CIS with a DVS enables the latency-tolerant restoration of motion-blurred images in mobile cameras. While prior event-based deblurring methods suffer from color ghosts, event noise, and discrepancies between CIS and DVS, we systematically analyzed these artifacts and introduced a set of event-driven compensation techniques: cross-correlation-based local threshold optimization, contrast maximization for noise suppression, event-based resolution mismatch compensation, and disparity- and timing-matching between the two sensors. Using a Dead Leaves chart and SFR analysis together with PSNR and SSIM, we showed that the proposed technique improves the restoration quality from 18.52 dB/0.683 to 38.72 dB/0.911 under realistic motion and low-illumination conditions, while remaining suitable for low-latency implementation on a commercial smartphone platform. These results indicate that event-based vision sensors can complement existing CIS architectures to deliver motion blur-resilient imaging without incurring prohibitive computational cost. Future work will focus on accelerating the correlation-driven optimization and event processing on mobile GPU/NPU/ISP blocks and extending the approach to more complex scenes and hybrid (learning-assisted) restoration.

## Figures and Tables

**Table 1:** Key characteristics of the CIS and the DVS used in this work.

Characteristics	Items	CIS (GM1)	DVS (RC1)
Specifications	Optical Format	1/2″	1/3.03″
	Resolution	4000 × 3000	960 × 720
	Pixel Pitch	0.8 μm	4.95 μm
	Frame Rate	30 fps	2000 fps (minimum)
Attributes	Sensing Principle	Frame-based integration of intensity over exposure	Event-based thresholding of log-intensity change
	Output	Full frames at fixed rate	Asynchronous event stream
	Shutter	Electronic rolling shutter	Global event holding
	Strengths	Photorealistic intensity/color	Motion-robust edge timing
	Role in our work	Provides the target image content	Provides motion/edge cues

**Table 2:** Quantitative summary for Figure 8: PSNR, SSIM, and SFR (MTF50 ratio).

	PSNR (dB)	SSIM	SFR (MTF50 Ratio)
Motion-blurred input (b)	18.52	0.683	0.39
Baseline EDI (c)	27.74	0.787	0.64
EDI w/compensation (d)	37.86	0.901	0.90
EDI w/compensation (e)	38.67	0.909	0.98
EDI w/compensation (f)	38.72	0.911	0.99

## Data Availability

Dataset available on request from the authors.

## Supplementary Materials

The following supporting information can be downloaded at: https://www.mdpi.com/article/10.3390/s26030781/s1, Figure S1: Pseudo-color visualization of the event-noise filtering effect for Figure 6. Two representative ROIs are shown before and after the proposed event-noise filtering. A pseudo-color palette is used to make the speckle-like noise pattern more visible than in grayscale, thereby clarifying that the filtering reduces isolated speckles and stabilizes the event representation for downstream restoration/fusion.

## Abbreviations

The following abbreviations are used in this manuscript:

CMOS	Complementary Metal-Oxide Semiconductor
CIS	CMOS Image Sensor
DVS	Dynamic Vision Sensor
fps	Frames Per Second
PSNR	Peak Signal-to-Noise Ratio
SSIM	Structural Similarity Index Measure
SFR	Spatial Frequency Response
FFT	Fast Fourier Transform
PSD	Power Spectral Density
GT	Ground Truth
MTF	Modulation Transfer Function
ESF	Edge Spread Function
ISO	International Organization for Standardization
LSF	Line Spread Function
BEW	Blurred Edge Width
VoL	Variance of Laplacian
EDI	Event-Based Double Integral
LP/PH	Line Pairs/Picture Height
CM	Contrast Maximization
HDR	High Dynamic Range
ISP	Image Signal Processor
SoC	System on Chip
