# Supplementary material for: High-Speed Image Restoration Based on a Dynamic Vision Sensor
Source: Sensors (Basel). 2026 Jan 23;26(3):781. doi: 10.3390/s26030781 (PMC12899907; doi:10.3390/s26030781)
Supplement: Supplementary file 1 [file sensors-26-00781-s001.zip › sensors-4068620-supplementary.pdf]

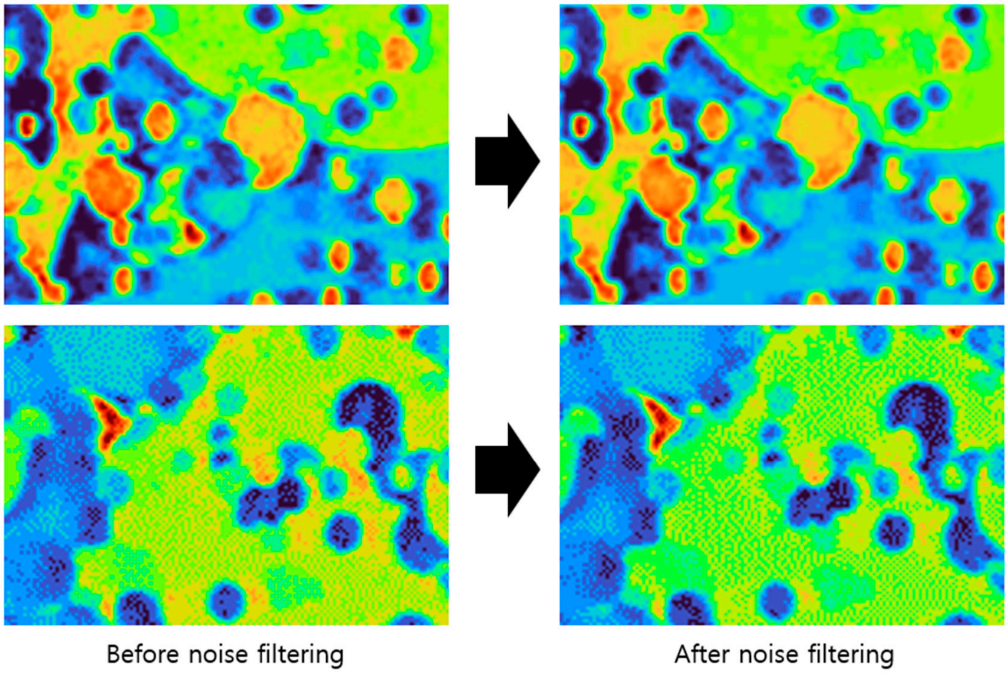

Figure S1. Pseudo-color visualization of the event-noise filtering effect for Fig. 6. Two representative ROIs are shown before and after the proposed event-noise filtering. A pseudo-color palette is used to make the speckle-like noise pattern more visible than in grayscale, thereby clarifying that the filtering reduces isolated speckles and stabilizes the event representation for downstream restoration/fusion.

Supplementary Material (Visualization for Figure 6).

To better illustrate the effect of the proposed event-noise filtering, we provide pseudo-color visualizations of representative ROIs from Fig. 6. In particular, a pseudo-color palette (e.g., turbo) is applied to the same ROI regions to emphasize the speckle-like pattern of spurious event noise and its suppression after filtering. This visualization is intended to facilitate qualitative inspection of noise accumulation reduction (which mainly benefits subsequent processing stages), rather than to claim an additional standalone quantitative benchmark.
